# Supplementary material for: A Likelihood Approach for Real-Time Calibration of Stochastic Compartmental Epidemic Models
Source: PLoS Comput Biol. 2017 Jan 17;13(1):e1005257. doi: 10.1371/journal.pcbi.1005257 (PMC5240920; doi:10.1371/journal.pcbi.1005257)
Supplement: S1 File — (TAR.GZ) [file pcbi.1005257.s014.tar.gz › HSPH_Online-SI-Revision/PF08/n1-extreme/n1-extreme_PF_table4.pdf]

1	R0	{2.95019, 14.2854}	{2.34391, 15.8207}	{{1.7174, 10.404}, {3., 20.}}	{20.9568, 23.2172}	inf	101.	122.044	{48.5532, 234.084}	46.1235	0.18144	newly	40.	47.8	{26., 85.}	39.2	0.146213	Reff	0.31272	0.60865	{0.364435, 0.858644}	94.6657	ar	924.	976.72	{822., 1075.}	11.658
2	R0	{2.67565, 16.946}	{2.09693, 15.5491}	{{1.46555, 11.7692}, {2.83271, 20.}}	{23.9081, 17.5383}	inf	81.	76.9934	{26.6881, 148.739}	43.0328	0.219992	newly	24.	31.86	{13., 53.}	58.25	0.213707	Reff	0.294322	0.632791	{0.357704, 0.937746}	115.	ar	926.	921.01	{695., 1049.}	10.7419
3	R0	{2.40066, 16.6543}	{2.09462, 15.4245}	{{1.33008, 10.2585}, {2.78471, 19.7235}}	{19.594, 16.7891}	inf	164.	95.5412	{31.4978, 181.684}	46.8716	0.331343	newly	51.	40.1	{14., 70.}	39.8039	0.237704	Reff	0.40091	0.738454	{0.420709, 1.04464}	84.5389	ar	916.	904.12	{612., 1051.}	12.7686
4	R0	{1.9294, 15.4361}	{2.02172, 14.9991}	{{1.28352, 8.66548}, {2.84054, 19.8941}}	{22.9044, 21.998}	inf	105.	75.6672	{19.2731, 163.019}	48.684	0.322548	newly	40.	30.9	{11., 51.}	41.2	0.24738	Reff	0.677219	0.692383	{0.394617, 0.990907}	25.8741	ar	796.	870.13	{553., 1045.}	21.7726
5	R0	{2.92859, 19.5393}	{1.71233, 15.6587}	{{1.24009, 9.27459}, {2.14971, 20.}}	{41.5794, 20.9866}	inf	114.	41.5567	{5.53748, 83.3664}	63.5468	0.610344	newly	40.	15.34	{3., 27.}	62.1	0.564255	Reff	0.351431	0.604216	{0.367324, 0.82472}	74.437	ar	927.	785.53	{542., 970.}	17.2977
6	R0	{1.79622, 12.3148}	{1.8864, 14.5242}	{{1.28574, 6.03821}, {2.52927, 20.}}	{21.9968, 36.3375}	inf	56.	50.367	{13.7852, 118.705}	56.9897	0.302571	newly	31.	22.29	{8., 42.}	48.0323	0.293578	Reff	0.661009	0.806647	{0.521128, 1.11245}	32.7578	ar	734.	815.43	{593., 1008.}	22.3147
7	R0	{2.45855, 17.1776}	{1.87656, 14.0065}	{{1.21457, 8.42466}, {2.73737, 19.0665}}	{27.6778, 22.6064}	inf	121.	55.1618	{9.5169, 94.5394}	59.0567	0.50838	newly	52.	23.07	{5., 39.}	58.2115	0.471737	Reff	0.486793	0.617699	{0.336191, 0.873888}	41.2326	ar	871.	694.16	{381., 908.}	22.124
8	R0	{2.20009, 15.519}	{1.82436, 15.1685}	{{1.28298, 8.97084}, {2.41512, 20.}}	{23.7448, 24.9442}	inf	65.	51.9365	{5.8082, 94.0816}	48.7398	0.357169	newly	25.	20.4	{3., 33.}	40.16	0.292061	Reff	0.448818	0.601011	{0.331567, 0.894241}	45.3994	ar	839.	830.19	{561., 1030.}	16.6377
9	R0	{2.06493, 9.6414}	{2.27793, 15.6207}	{{1.62682, 11.3315}, {3., 19.5207}}	{22.8991, 64.2756}	inf	54.	106.472	{36.6844, 181.125}	106.43	0.289917	newly	29.	43.81	{21., 76.}	62.6552	0.197403	Reff	0.580245	0.708526	{0.448803, 1.00847}	35.7178	ar	777.	946.51	{783., 1077.}	24.7246
10	R0	{2.60775, 19.6936}	{1.703, 14.8756}	{{1.19502, 8.89231}, {2.24515, 20.}}	{35.2686, 25.0141}	inf	139.	47.0086	{7.0337, 87.3122}	66.7193	0.634714	newly	45.	19.05	{5., 34.}	58.5111	0.499551	Reff	0.47461	0.628981	{0.365357, 0.875611}	42.0034	ar	889.	778.65	{548., 985.}	17.315
11	R0	{1.99168, 8.13555}	{2.13783, 14.5082}	{{1.37169, 8.23597}, {2.93093, 20.}}	{23.9741, 80.1696}	inf	45.	70.3158	{19.5159, 116.313}	87.2915	0.275804	newly	31.	29.5	{12., 53.}	43.6129	0.205556	Reff	0.579579	0.703939	{0.43382, 1.00523}	34.752	ar	772.	884.7	{663., 1046.}	21.0933
12	R0	{1.79304, 9.18941}	{2.11646, 14.8275}	{{1.5278, 7.33552}, {2.79528, 19.4184}}	{26.237, 69.2047}	inf	52.	108.466	{32.4809, 218.97}	122.786	0.322155	newly	36.	46.76	{23., 85.}	48.8333	0.173853	Reff	0.627564	0.808232	{0.478618, 1.18217}	38.4886	ar	725.	908.38	{726., 1069.}	29.0759
13	R0	{1.84016, 18.3431}	{1.67044, 14.8853}	{{1.20924, 8.12987}, {2.2747, 20.}}	{22.5374, 22.5603}	inf	88.	39.3974	{9.16198, 77.2846}	57.1684	0.492608	newly	32.	15.82	{5., 26.}	53.25	0.41778	Reff	0.875916	0.640652	{0.348469, 0.889906}	28.4528	ar	762.	760.6	{508., 989.}	20.5748
14	R0	{1.94221, 9.85971}	{2.11519, 15.1433}	{{1.41502, 7.38867}, {2.97814, 20.}}	{23.7343, 61.4211}	inf	31.	75.547	{18.8291, 144.262}	158.721	0.387591	newly	20.	30.3	{12., 51.}	72.4	0.238521	Reff	0.42146	0.621298	{0.35728, 0.919065}	54.7859	ar	805.	899.58	{637., 1060.}	20.6236
15	R0	{1.78578, 12.0541}	{1.64237, 14.5986}	{{1.20039, 7.40303}, {2.30451, 20.}}	{20.9652, 40.0328}	inf	61.	42.1945	{4.8979, 76.4538}	51.5932	0.445313	newly	32.	16.62	{4., 30.}	52.875	0.474875	Reff	0.558949	0.651571	{0.390111, 0.873036}	32.1112	ar	732.	742.49	{486., 963.}	21.0232
16	R0	{1.94463, 15.6503}	{1.82187, 13.6869}	{{1.18406, 6.8071}, {2.46976, 18.7084}}	{19.7603, 22.6991}	inf	82.	60.6212	{5.17802, 113.621}	50.277	0.407108	newly	29.	26.23	{3., 53.}	51.8966	0.353881	Reff	0.663119	0.681809	{0.371584, 0.964316}	28.3245	ar	757.	673.33	{290., 883.}	20.3633
17	R0	{2.08901, 16.4314}	{1.74184, 14.8138}	{{1.24295, 7.75348}, {2.40398, 20.}}	{24.7285, 22.1952}	inf	116.	65.4383	{16.692, 139.752}	51.7155	0.389719	newly	42.	27.52	{10., 49.}	44.619	0.291996	Reff	0.676839	0.720433	{0.378446, 1.03805}	31.1619	ar	826.	789.3	{553., 1040.}	18.1041
18	R0	{2.76419, 18.56}	{2.11969, 15.3812}	{{1.51183, 11.2245}, {2.89861, 20.}}	{25.1135, 19.9217}	inf	153.	102.116	{38.4674, 194.847}	46.4684	0.293415	newly	51.	42.25	{21., 74.}	39.3529	0.209697	Reff	0.351052	0.722089	{0.407518, 0.974372}	105.731	ar	920.	909.04	{721., 1054.}	11.513
19	R0	{2.3821, 17.1399}	{1.95356, 13.706}	{{1.34861, 7.10524}, {2.76795, 19.1609}}	{25.0333, 24.9827}	inf	162.	70.2357	{9.60446, 137.136}	60.9967	0.558011	newly	73.	29.94	{7., 60.}	61.2877	0.52568	Reff	0.443071	0.657942	{0.325052, 1.00575}	59.7436	ar	883.	713.8	{439., 915.}	20.6727
20	R0	{2.78314, 19.6127}	{1.80907, 14.3872}	{{1.29288, 7.66917}, {2.40725, 20.}}	{35.1549, 27.4188}	inf	71.	43.469	{6.3099, 94.1818}	52.7321	0.413752	newly	24.	18.04	{4., 31.}	45.9167	0.31883	Reff	0.222651	0.58568	{0.330685, 0.840795}	163.15	ar	943.	818.91	{555., 1029.}	17.5451
21	R0	{1.85124, 14.6794}	{1.72676, 14.7395}	{{1.25082, 8.27042}, {2.36846, 19.9974}}	{20.9036, 22.6583}	inf	108.	63.4569	{12.3899, 137.426}	55.5151	0.421848	newly	46.	25.76	{7., 50.}	50.6957	0.371284	Reff	0.794182	0.720623	{0.41798, 1.01441}	24.6042	ar	730.	787.19	{527., 1003.}	21.2397
22	R0	{2.1028, 14.9852}	{2.0741, 15.1363}	{{1.41021, 9.56464}, {2.74368, 20.}}	{21.1886, 20.0987}	inf	84.	68.9983	{21.5069, 137.736}	45.1457	0.260045	newly	32.	29.2	{13., 56.}	44.125	0.223326	Reff	0.471027	0.713943	{0.414191, 1.06149}	56.1951	ar	840.	878.67	{640., 1036.}	16.1583
23	R0	{1.86601, 10.2157}	{1.87039, 14.0477}	{{1.34091, 8.39294}, {2.45256, 18.8228}}	{18.5965, 45.0307}	inf	60.	67.0247	{7.72364, 128.542}	60.4523	0.322712	newly	37.	28.52	{6., 54.}	46.5405	0.317801	Reff	0.649371	0.660376	{0.419175, 0.88956}	25.6739	ar	747.	701.5	{443., 879.}	17.7697
24	R0	{2.34032, 10.0992}	{2.2604, 14.9352}	{{1.59206, 10.5531}, {2.99553, 20.}}	{17.1256, 51.2209}	inf	58.	96.8563	{44.1797, 150.172}	76.374	0.233137	newly	35.	41.62	{22., 63.}	38.4	0.146471	Reff	0.355729	0.607268	{0.352119, 0.948503}	73.1197	ar	887.	959.5	{791., 1068.}	12.7982
25	R0	{1.91548, 15.3401}	{1.877, 15.0638}	{{1.27376, 9.39749}, {2.44531, 19.9038}}	{19.3163, 21.3739}	inf	102.	75.1733	{24.7496, 154.87}	44.6401	0.279686	newly	45.	31.72	{14., 55.}	41.1111	0.24745	Reff	0.739375	0.797361	{0.519385, 1.07884}	24.7167	ar	765.	830.35	{560., 1022.}	19.8941
26	R0	{2.75283, 10.6721}	{2.34722, 14.632}	{{1.58702, 9.29067}, {3., 19.9821}}	{18.477, 45.9815}	inf	40.	101.791	{35.4599, 175.123}	161.184	0.383116	newly	22.	41.66	{23., 60.}	93.	0.263879	Reff	0.286294	0.557114	{0.328706, 0.819071}	94.5947	ar	926.	962.26	{748., 1065.}	11.6739
27	R0	{2.08367, 19.7849}	{1.75592, 14.7154}	{{1.2465, 7.91549}, {2.36652, 19.9791}}	{21.4643, 25.861}	inf	132.	51.2658	{9.69338, 104.956}	62.8729	0.569178	newly	42.	20.18	{6., 36.}	54.0952	0.457909	Reff	0.614683	0.661606	{0.385861, 0.906169}	29.217	ar	833.	808.54	{549., 1009.}	16.9868
28	R0	{2.64334, 17.1765}	{2.18615, 15.6901}	{{1.58691, 10.2009}, {2.88966, 20.}}	{21.1008, 17.7748}	inf	110.	75.4244	{25.3166, 115.97}	42.3829	0.278945	newly	34.	30.24	{14., 47.}	37.1176	0.191438	Reff	0.311914	0.656729	{0.414637, 0.93101}	110.548	ar	934.	913.91	{733., 1047.}	10.6135
29	R0	{2.87008, 13.3872}	{2.04497, 15.9726}	{{1.33628, 10.5075}, {2.91013, 20.}}	{29.4955, 30.3306}	inf	80.	74.4835	{21.3018, 124.21}	42.7685	0.238176	newly	36.	28.04	{11., 43.}	33.1667	0.207054	Reff	0.226736	0.541308	{0.259139, 0.841001}	139.154	ar	941.	886.05	{618., 1050.}	12.8087
30	R0	{2.06105, 19.6275}	{1.68454, 14.2248}	{{1.22812, 7.77108}, {2.29733, 20.}}	{24.7237, 28.0117}	inf	42.	26.0738	{1.93989, 54.2982}	55.8154	0.483358	newly	18.	10.46	{2., 22.}	52.2222	0.449841	Reff	0.595643	0.605267	{0.333493, 0.796311}	25.897	ar	747.	767.25	{532., 995.}	20.8929
31	R0	{2.30764, 13.435}	{2.19891, 15.5202}	{{1.54571, 10.1284}, {2.95063, 19.7477}}	{19.7195, 27.8164}	inf	82.	96.6373	{32.4592, 177.139}	49.8566	0.20695	newly	42.	39.58	{19., 61.}	34.1905	0.166044	Reff	0.376145	0.676421	{0.434775, 0.966746}	79.9268	ar	877.	924.41	{731., 1073.}	14.0171
32	R0	{2.30565, 11.9237}	{2.16375, 15.0687}	{{1.43984, 11.0461}, {2.8611, 19.645}}	{18.3178, 33.3613}	inf	65.	94.1165	{33.4101, 180.253}	66.9091	0.228732	newly	34.	39.24	{20., 62.}	39.4706	0.154959	Reff	0.378127	0.706915	{0.4319, 1.06793}	86.952	ar	853.	919.86	{723., 1059.}	15.8382
33	R0	{2.24031, 18.582}	{1.73231, 15.3343}	{{1.25948, 9.60465}, {2.16692, 20.}}	{26.6164, 21.0086}	inf	156.	69.803	{21.8062, 139.222}	57.9159	0.477488	newly	47.	27.64	{11., 51.}	47.1064	0.341213	Reff	0.578	0.684845	{0.394028, 0.895403}	31.5858	ar	864.	799.36	{563., 967.}	15.0694
34	R0	{2.33306, 15.9759}	{2.05222, 14.9203}	{{1.38555, 7.7408}, {2.83359, 20.}}	{20.2861, 22.7424}	inf	110.	86.0979	{25.5108, 172.312}	47.0814	0.300794	newly	38.	35.07	{13., 60.}	43.0263	0.219145	Reff	0.468945	0.738393	{0.449504, 0.977233}	59.8652	ar	854.	885.58	{638., 1050.}	15.9578
35	R0	{2.48357, 18.3573}	{2.04905, 14.8056}	{{1.45504, 6.59437}, {2.74097, 20.}}	{22.348, 23.4827}	inf	120.	80.9783	{22.6413, 138.92}	44.6866	0.302393	newly	42.	34.89	{17., 61.}	39.2619	0.214975	Reff	0.588606	0.81703	{0.490816, 1.09219}	45.1783	ar	835.	872.32	{633., 1034.}	16.3042
36	R0	{2.59548, 19.9046}	{2.05323, 14.6439}	{{1.40271, 7.21418}, {2.78932, 20.}}	{24.3076, 26.5552}	inf	123.	75.1625	{18.6854, 160.414}	53.4978	0.39733	newly	46.	30.94	{9., 61.}	47.2609	0.315718	Reff	0.490546	0.682979	{0.428317, 0.96578}	45.1165	ar	863.	881.56	{624., 1046.}	15.8934
37	R0	{2.29641, 16.9868}	{1.74313, 14.8549}	{{1.2916, 7.74374}, {2.32007, 20.}}	{26.1379, 20.7821}	inf	111.	37.3832	{6.7405, 84.2444}	66.4481	0.68677	newly	31.	14.8	{3., 28.}	55.6774	0.509059	Reff	0.420243	0.587327	{0.319006, 0.800465}	48.0755	ar	877.	788.32	{500., 980.}	16.4059
38	R0	{2.65743, 15.4569}	{1.96474, 15.2387}	{{1.36475, 7.80306}, {2.6242, 19.775}}	{27.5109, 19.9037}	inf	85.	75.3269	{26.4409, 158.285}	46.0835	0.247037	newly	40.	31.28	{13., 52.}	40.95	0.232926	Reff	0.385327	0.697932	{0.413683, 0.952739}	82.5976	ar	897.	876.96	{627., 1036.}	13.3244
39	R0	{2.67953, 14.5132}	{2.09996, 15.2798}	{{1.46044, 7.4724}, {2.86653, 20.}}	{24.5861, 25.7347}	inf	119.	95.5916	{31.0709, 179.06}	41.2217	0.245709	newly	46.	39.78	{17., 67.}	37.1739	0.200925	Reff	0.404609	0.744779	{0.420174, 1.15229}	85.4857	ar	894.	911.55	{727., 1058.}	12.4083
40	R0	{1.94594, 11.928}	{1.88792, 14.0979}	{{1.31748, 8.26611}, {2.46976, 19.255}}	{19.2747, 34.1551}	inf	60.	46.338	{2.82989, 112.742}	58.2112	0.468071	newly	31.	18.87	{2., 42.}	56.6129	0.48095	Reff	0.558485	0.593613	{0.318905, 0.834741}	31.1377	ar	796.	705.02	{444., 890.}	17.804
41	R0	{1.66297, 10.0047}	{1.65945, 15.3342}	{{1.18522, 9.39342}, {2.35373, 20.}}	{20.7013, 59.2283}	inf	53.	42.6166	{6.77954, 80.5486}	52.6514	0.368519	newly	33.	16.56	{4., 32.}	53.0303	0.444555	Reff	0.640243	0.659954	{0.356952, 0.898447}	27.8461	ar	716.	765.23	{510., 998.}	22.655
42	R0	{2.91568, 16.4453}	{2.22465, 14.7491}	{{1.56069, 9.92109}, {2.98476, 20.}}	{24.3248, 20.4831}	inf	138.	100.857	{39.3005, 162.794}	35.8769	0.236607	newly	55.	43.22	{18., 73.}	36.1091	0.21028	Reff	0.297399	0.653502	{0.382639, 0.936217}	119.739	ar	919.	947.37	{771., 1052.}	10.3536
43	R0	{2.55898, 15.8889}	{2.04256, 14.5406}	{{1.37826, 7.15893}, {2.77813, 19.6364}}	{24.121, 21.0586}	inf	147.	84.5703	{28.248, 187.601}	53.0512	0.373261	newly	62.	37.39	{15., 65.}	47.6935	0.321241	Reff	0.455498	0.788641	{0.466893, 1.127}	75.0506	ar	904.	879.92	{636., 1051.}	13.3606
44	R0	{2.28649, 15.9017}	{2.13502, 15.6723}	{{1.46178, 10.5983}, {2.78507, 20.}}	{19.5343, 17.3479}	inf	92.	80.7284	{32.1158, 157.255}	43.1562	0.229091	newly	43.	32.8	{14., 58.}	40.7442	0.24158	Reff	0.400136	0.666093	{0.379835, 1.01525}	68.6568	ar	880.	921.44	{692., 1059.}	13.9636
45	R0	{2.65121, 18.6339}	{2.19328, 14.5674}	{{1.62558, 10.3431}, {2.77093, 19.4683}}	{19.7537, 23.5574}	inf	160.	93.8758	{36.9338, 147.813}	47.7625	0.322719	newly	45.	40.44	{16., 64.}	36.0889	0.188908	Reff	0.371169	0.716783	{0.454702, 1.03221}	93.5025	ar	928.	923.85	{788., 1051.}	9.48168
46	R0	{2.52291, 18.0251}	{2.1008, 15.3316}	{{1.383, 9.83601}, {2.7717, 20.}}	{20.4154, 19.3529}	inf	131.	86.0988	{29.0974, 174.836}	48.159	0.326907	newly	46.	36.25	{13., 69.}	40.8043	0.24143	Reff	0.431418	0.677944	{0.415004, 0.975033}	59.4059	ar	894.	909.93	{664., 1040.}	12.5224
47	R0	{2.22618, 17.1225}	{2.02012, 14.8452}	{{1.27529, 9.73678}, {2.89326, 19.8197}}	{22.1501, 20.7686}	inf	148.	75.93	{27.2198, 147.271}	53.8012	0.40225	newly	59.	32.91	{13., 61.}	50.1864	0.342774	Reff	0.634461	0.800925	{0.483416, 1.1601}	38.8146	ar	850.	864.33	{559., 1051.}	17.5094
48	R0	{2.04405, 16.073}	{1.87422, 13.6714}	{{1.22544, 7.10524}, {2.59755, 19.0269}}	{21.8704, 24.9431}	inf	103.	54.7845	{6.20046, 123.823}	59.8198	0.564948	newly	40.	23.67	{4., 49.}	58.375	0.484413	Reff	0.711329	0.625301	{0.32281, 0.888148}	27.1839	ar	779.	695.74	{412., 902.}	19.3196
49	R0	{2.84396, 18.097}	{2.30509, 14.7282}	{{1.57153, 9.05622}, {2.92106, 19.1521}}	{20.1981, 21.3003}	inf	132.	85.4886	{35.8137, 143.548}	41.9199	0.276923	newly	43.	36.57	{17., 55.}	31.6977	0.171279	Reff	0.264488	0.597616	{0.40191, 0.884167}	125.952	ar	952.	941.02	{753., 1059.}	9.72899
50	R0	{1.85341, 18.0975}	{1.85201, 13.7732}	{{1.21725, 7.29751}, {2.48103, 18.3794}}	{22.2196, 25.0006}	inf	90.	46.6574	{3.56478, 89.2441}	58.0879	0.548851	newly	26.	19.75	{3., 36.}	51.6538	0.403192	Reff	0.615332	0.615106	{0.325206, 0.893132}	30.6777	ar	754.	692.82	{402., 890.}	19.7427
